# Efficacy of Qigong Exercise for Treatment of Fatigue: A Systematic Review and Meta-Analysis

**DOI:** 10.3389/fmed.2021.684058

**Published:** 2021-06-22

**Authors:** Rui Wang, Xueyan Huang, Yeqi Wu, Dai Sun

**Affiliations:** ^1^Department of Massage, Hangzhou TCM Hospital Affiliated to Zhejiang Chinese Medical University, Hangzhou, China; ^2^Third Clinical Medical College, Zhejiang Chinese Medical University, Hangzhou, China

**Keywords:** qigong exercise, fatigue, quality of life, systematic review, meta-analysis

## Abstract

**Objective:** Several studies suggested that Qigong exercise (QE) can relieve fatigue in patients diagnosed with various diseases. Our review aimed to evaluate the efficacy of QE for alleviating fatigue.

**Methods:** A related literature search was performed in the PubMed, Web of Science, Embase, Cochrane Library, China Biology Medicine disc (CBM), China National Knowledge Infrastructure (CNKI), Wanfang, and VIP data bases from inception to November 2020. Information on fatigue, malaise, tiredness, and Qigong research data was collected.

**Results:** Sixteen randomized controlled trials (RCTs) were reported in patients with cancer (*n* = 4), chronic fatigue syndrome (*n* = 2), and other diseases (*n* = 10). The QE groups showed significant improvements in total fatigue intensity [15 RCTs, *p* < 0.00001; standard mean difference (SMD) −0.69 (−0.95 to −0.44)]. The QE groups did not show significant improvement in quality of life [4 RCTs, *p* = 0.08; SMD 0.53 (−0.07 to 1.14)]. The statistically significant difference of the subgroup analyses (different primary diseases, QE types, and study quality) also remained unchanged.

**Conclusion:** The findings of this meta-analysis indicate that QE may be beneficial for improving fatigue in patients diagnosed with various diseases. Considering the limitations of the study, we draw a very cautious conclusion regarding the resulting estimate of the effect. Further studies are warranted to better understand the benefits of QE in primary medical care.

## Introduction

Fatigue, a subjective symptom, is the embodiment of complex physiological, psychological, and pathological phenomena, wherein the body's physical strength or energy is weakened or lost (1). With the rapid industrialization of society, chronic fatigue is prevalent in 15–20% of the general population (2, 3). In some occupations or specific environments, the incidence rate of fatigue symptoms is even >70% (4, 5). Fatigue is also one of the most common symptoms in primary medical care. Approximately 10–20% of patients complain of fatigue (6, 7), and 10–30% of patients consider fatigue an important accompanying symptom (8, 9). Several drugs, such as anti-neoplastic agents (10) and antidepressants (11), may be associated with secondary fatigue, which is produced by the adverse effects of the administered treatment. For the general population, fatigue is an alarm signal given by the body when it attains its physical limits. Without effective treatment, the symptoms of physical illness and common psychological disorders may lead patients into a vicious cycle associated strongly with fatigue, depression, anxiety, chronic pain, and sleep disturbances (12). However, clinically, patients describe fatigue in varying ways, such as sleepiness, weakness, lethargy, pain, and heaviness, and physicians may overlook or underestimate its severity (13).

The treatment options for fatigue should address the etiology and mechanism of the primary diseases, if possible. However, the multidimensionality and subjective nature of fatigue, and the lack of consensus on its pathophysiology make effective treatment difficult (14). As a result, a standardized and specific treatment that may improve or prevent fatigue in population groups that are considered “patients at risk” has not been established (15, 16). Methods for improving fatigue in patients are divided into pharmacological and non-pharmacological categories. However, some drugs used for treating fatigue possess insufficient evidence of efficacy and should be further investigated (17, 18); the adverse effects of other medications could cause more complications in patients (19, 20). Aerobic exercise has been recommended as a non-pharmacological alternative for treating fatigue (21, 22). It is a practical option and can easily be adhered to by patients in the long-term. Unfortunately, the efficacy of aerobic exercise in improving fatigue has also been questioned in some systematic reviews (23, 24).

Qigong exercise (QE), a distinctive traditional technique that has amassed a large following in China, is a form of low-intensity exercise (25, 26). Numerous clinical guidelines related to the use of QE for the rehabilitation of fatigue in patients with cardiopulmonary diseases and Parkinson's disease are available, with positive recommendations (27, 28). Other systematic reviews either evaluated QE as a component of aerobic exercise or aimed to summarize the potential of QE to address the multifaceted needs of cancer survivors (21, 22). In this study, we aimed to comprehensively evaluate the therapeutic benefits of QE on fatigue. We investigated a broader scope of primary diseases and patient clinical outcomes by summarizing and analyzing the existing literature.

## Methods

This systematic review was performed in accordance with the Preferred Reporting Items for Systematic Reviews and Meta-Analyses (PRISMA) guideline (Supplementary Appendix 1). The study had been registered on INPLASY (https://inplasy.com) and the registration number is INPLASY2020110133. There was no direct patient or public involvement in this review.

### Data Sources and Searches

We searched the related studies from PubMed, Web of Science, Embase, Cochrane Library, China Biology Medicine disc, China National Knowledge Infrastructure, Wanfang, and VIP Data Knowledge Service Platform published from inception until November 2020.

Exhaustive search strategies for each electronic database were developed by the review team members. The English-language search strategy containing relevant terms was based on the concepts: (1) “Fatigue” or “Malaise” or “Tiredness,” (2) “Qigong,” and (3) “Randomized Clinical Trial.” In the Chinese databases, we used equivalent search terms. Search tactics for PubMed are shown in Supplementary Appendix 2.

### Inclusion and Exclusion Criteria

Studies considered in this meta-analysis were required to meet the following inclusion criteria. First, studies published in English or Chinese up to November 30, 2020 with a randomized controlled trial (RCT) design investigating the use of QE interventions for fatigue symptoms were considered for inclusion. Studies were included if appropriate data were available for the calculation of effect sizes in each treatment arm. Second, studies involving patients with primary disease-related symptoms of fatigue were included, regardless of the underlying disease, age, sex, education, ethnicity, and occupation of the studied patients. If the patients experienced serious complications, such as cognitive communication disorders, the study was excluded. Third, studies were included if QE alone (any form of Qigong category, including Baduanjin, Yijinjing, and Wuqinxi) or QE combined with other treatments was the main intervention in the observation group. No limit was set on the duration and frequency of treatment. Moreover, in the control group, the patients were treated with any type of intervention, including exercise, stretching, or sham Qigong; studies with observation groups were also included. Finally, the primary outcome defined to be compared was the severity, duration, frequency, or improvement of fatigue, evaluated through the following scales: Functional Assessment Chronic Illness Therapy–Fatigue, Chalder's Fatigue Scale, Multidimensional Fatigue Inventory-20, Fatigue Scale-14, Visual Analog Scales for fatigue, Fatigue Severity Scale, or Revised Piper fatigue Scale, and medical scales for some specific diseases including Functional Assessment of Cancer Therapy-General, 16-item Parkinson Fatigue Scale, or Fatigue scale in the European Organization for Research and Treatment of Cancer Quality of Life Questionnaire - Core 30. Other secondary outcomes were quality of life (QOL), assessed through the MOS item short-form health survey-36 and Medical Outcomes Study 12-Item Short-Form Health Survey.

### Study Selection and Data Extraction

When the results of one study were presented in several publications, only the most recent publication and complete data were considered. The eligibility of trials was assessed by two authors independently (WR and W-YQ). The following information was extracted by two authors (WR and H-XY) based on a fixed protocol: geographical location, primary disease, number of QE group and control participants, interventions of QE type, duration, and outcomes results. Discrepancies were settled by agreement, and a third author (SD) was consulted if necessary.

### Risk of Bias Assessment

Two researchers (WR and H-XY) independently assessed the quality of the selected studies according to the Cochrane Collaboration's tool for randomized controlled trials (29). Items were evaluated in three categories: low risk of bias, unclear bias, and high risk of bias. The following characteristics were evaluated: random sequence generation (selection bias), allocation concealment (selection bias), blinding of participants and personnel (performance bias), incomplete outcome data (attrition bias), selective reporting (reporting bias), and other biases resulting from these questions were graphed and assessed using Review Manager 5.4. Any disagreements were resolved by discussion or with the help of a third author (SD).

### Data Synthesis and Statistical Analysis

The traditional pair-wise efficacy data in populations were synthesized and statistically analyzed using Review Manager 5.4 by random effects modeling (weighted by the inverse of the variance). For continuous data including the total fatigue intensity and QOL scale score, which were measured using different tools, the standard mean difference (SMD) and 95% confidence intervals were used for effective evaluation. To ensure uniformity of the included data, all of them were used in the intention-to-treat analysis. *P*-values <0.05 were considered statistically significant unless otherwise specified.

Between-study heterogeneity was assessed using the χ^2^ and I^2^ tests. According to the Cochrane handbook, an I^2^ value > 60% was considered substantial, 30 −60% moderate, and <30% non-important heterogeneity (30). An I^2^ statistics of 50% or higher indicated the presence of heterogeneity. To further assess the influence of heterogeneity on the meta-analyses' conclusions, meta-regression and subgroup analysis were performed to assess the primary outcome data according to the primary diseases, QE type, sample size, year of publication, intervention length, and study quality (depending on whether random sequence generation and allocation concealment are used to determine the high or low quality of the studies). Visual inspection of asymmetry in funnel plots was conducted. Egger-weighted regression method using STATA 15.0 (Stata-Corp) was also used to statistically assess the publication bias.

## Results

### Studies Included in the Meta-Analysis

**Figure 1:**
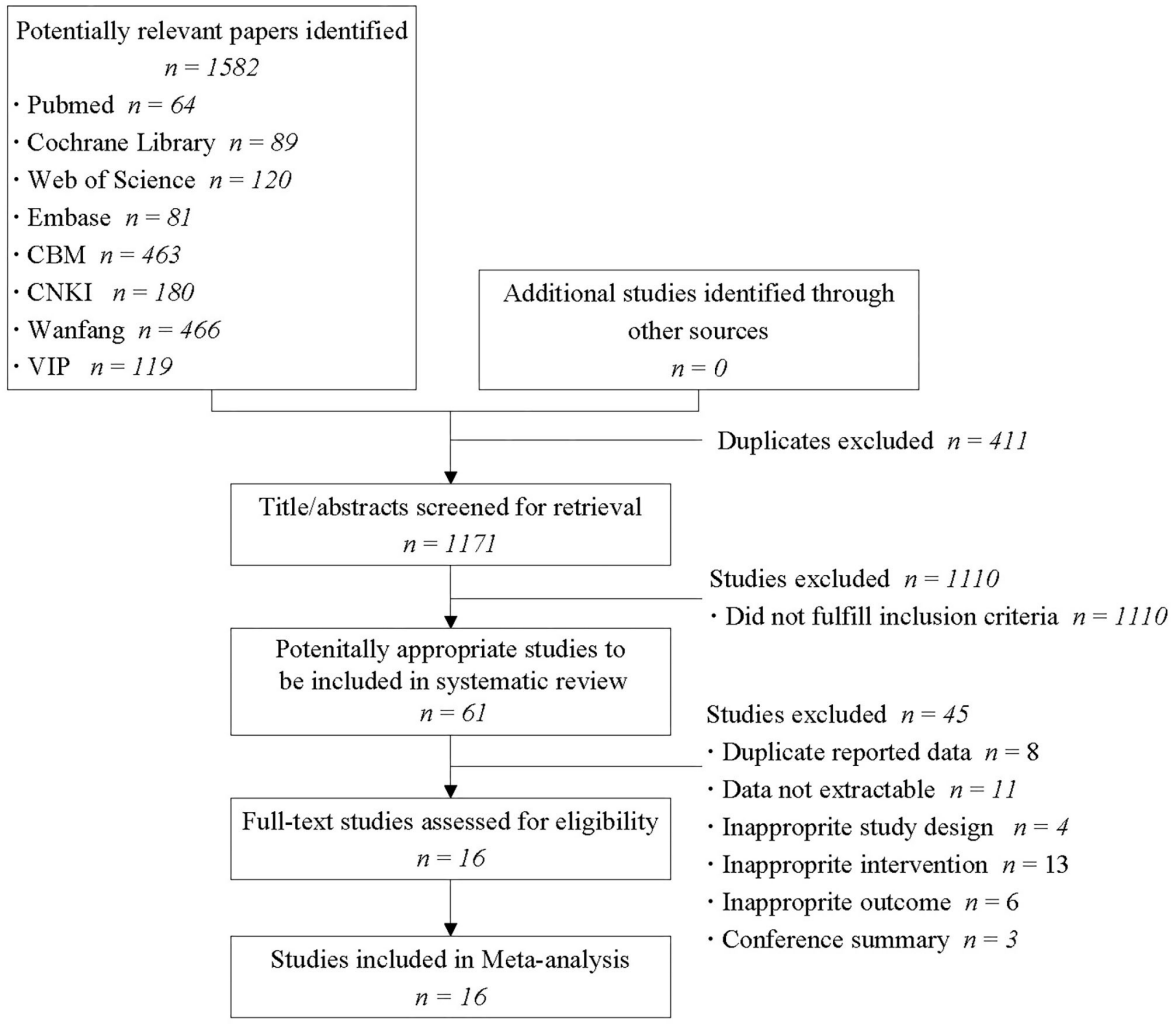
Flow diagram of the study selection process. CBM, Chinese biomedical literature database; CNKI, China national knowledge infrastructure.

Figure 1 presents a flow chart of the study selection process. Following the initial literature review, a total of 1,836 studies were found. Of these, 1,820 were excluded as only 16 studies met the inclusion criteria (Table 1) (31–46). The total number of participants included was 1,313, and the concise summary characteristics of each study are presented in Table 1. Of the 16 published studies, 11 were conducted in China, 4 in the United States, and 1 in Australia. Among these studies, the primary disease was cancer in 4 studies, chronic fatigue syndrome in 6, fibromyalgia in 2, post-stroke in 1, chronic obstructive lung disease in the stable phase in 1, inflammatory bowel disease in 1, and Parkinson's disease in 1. Sample sizes ranged from 14 to 162, and drop-out rates ranged from 0 to 33.33%.

**Table 1:** Characteristics of the included studies.

**References**	**Location**	**Primary disease**	**QE group N**	**Control group N**	**QE type**	**Duration (weeks)**	**Primary outcomes**
Campo et al., (45)	The United States	Cancer	20	20	Self-improving QE	12	Functional assessment chronic illness therapy–fatigue.
Chan et al., (32)	China	Chronic fatigue syndrome	72	65	Wu Xing Ping Heng Gong	17	Chalder's fatigue scale.
Chan et al., (33)	China	Chronic fatigue syndrome	75	75	Baduanjin	9	Chalder's fatigue scale1.
Duan (31)	China	Chronic fatigue syndrome	27	30	Shaolin QE	12	Multidimensional fatigue inventory-20, the MOS item short from health survey-36.
Lee, (34)	China	Cancer	16	29	Self-improving QE	12	Functional assessment of cancer therapy-general.
Lee, 2018b (34)			15	30			
Liu et al., (35)	The United States	Fibromyalgia	8	6	“Six healing sound” Qigong	6	Multidimensional fatigue inventory-20.
Moon et al., (36)	The United States	PD	8	9	“Six healing sound” Qigong	12	16-item parkinson fatigue scale.
Na et al., (37)	China	Chronic fatigue syndrome	15	30	Wuqinxi	4	Fatigue scale-14.
Na et al., (37)			15	15			
Na et al., (37)			30	15			
Oh et al., (38)	Australia	Cancer	79	83	Self-improving QE	10	Functional assessment of cancer therapy-general.
Rainbow, (44)	China	Chronic fatigue syndrome	33	31	Wu Xing Ping Heng Gong	17	Chalder's fatigue scale, medical outcomes study 12-item short-form health survey.
Sarmento et al., (39)	The United States	Fibromyalgia	10	10	“Six healing sound” Qigong	10	Visual analog scales for fatigue.
Tang et al., (40)	China	Inflammatory bowel disease	31	31	Baduanjin	12	Multidimensional fatigue inventory-20.
Wang, (41)	China	Post-stroke	30	30	Baduanjin	4	Fatigue severity scale.
Xu, (42)	China	Cancer	46	46	Baduanjin	26	Fatigue scale in the European organization for research and treatment of cancer quality of life questionnaire- core 30.
Yu et al., (43)	China	Chronic fatigue syndrome	21	21	Yijinjing	6	Fatigue scale-14.
Yu et al., (43)			21	42			
Yu et al., (43)			42	21			
Zhang, (44)	China	COPD	30	30	Baduanjin	26	Revised piper fatigue scale.

### Quality of Studies

**Figure 2:**
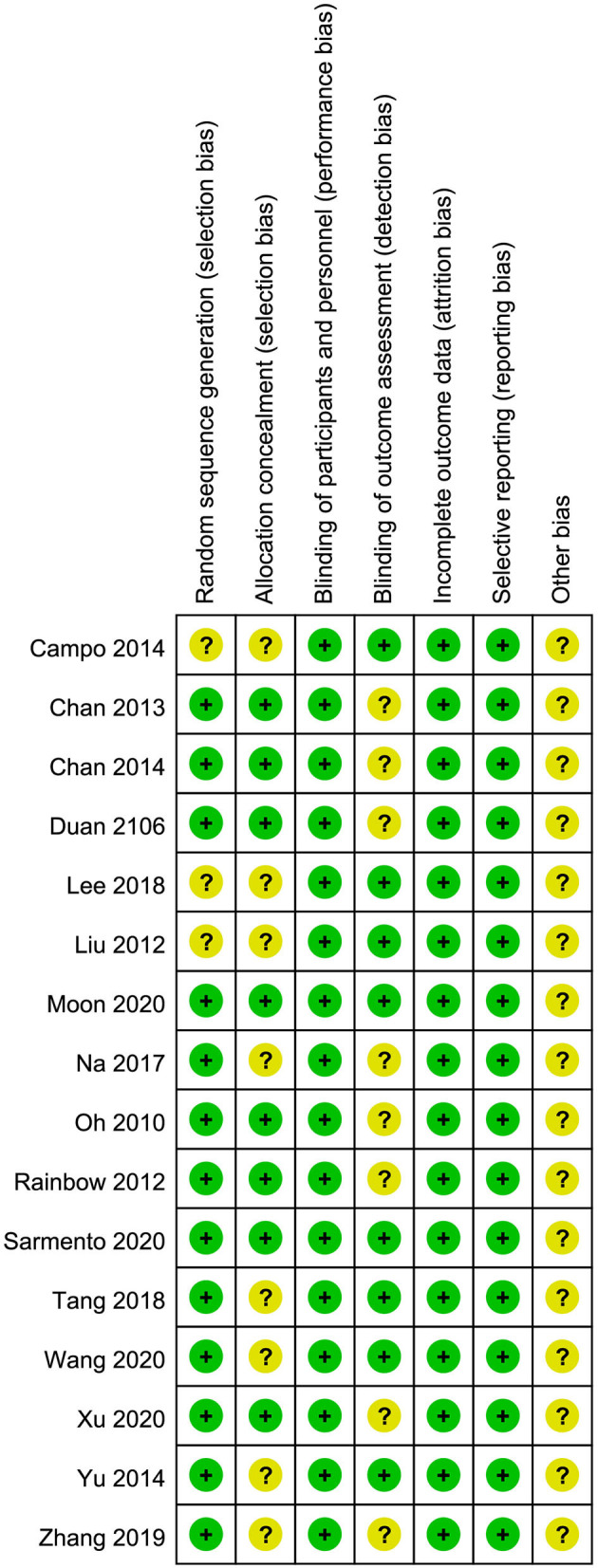
Risk of bias summary.

The quality of each study was evaluated considering certain methodological aspects by two authors (WR and H-XY) and is summarized in Figure 2. The generation of random sequences was adequate in 13 trials and unclear in the remaining trials. Allocation concealment was judged to be sufficient to minimize the selection bias in 8 trials; however, it was unclear in other trials. In particular, as blinding could not be applied to both participants and researchers owing to the nature of QE, blinding was judged adequate to prevent performance bias in all trials. Blinding of the outcome assessment was considered sufficient to prevent detection bias in eight trials and was unclear in eight trials. In all the trials, the quality of incomplete outcome data was adequate. No selective reporting of outcomes was observed, and other biases were classified as unclear.

### Evidence From Randomized Trials

#### Primary Outcomes

##### Total Fatigue Intensity

**Figure 3:**
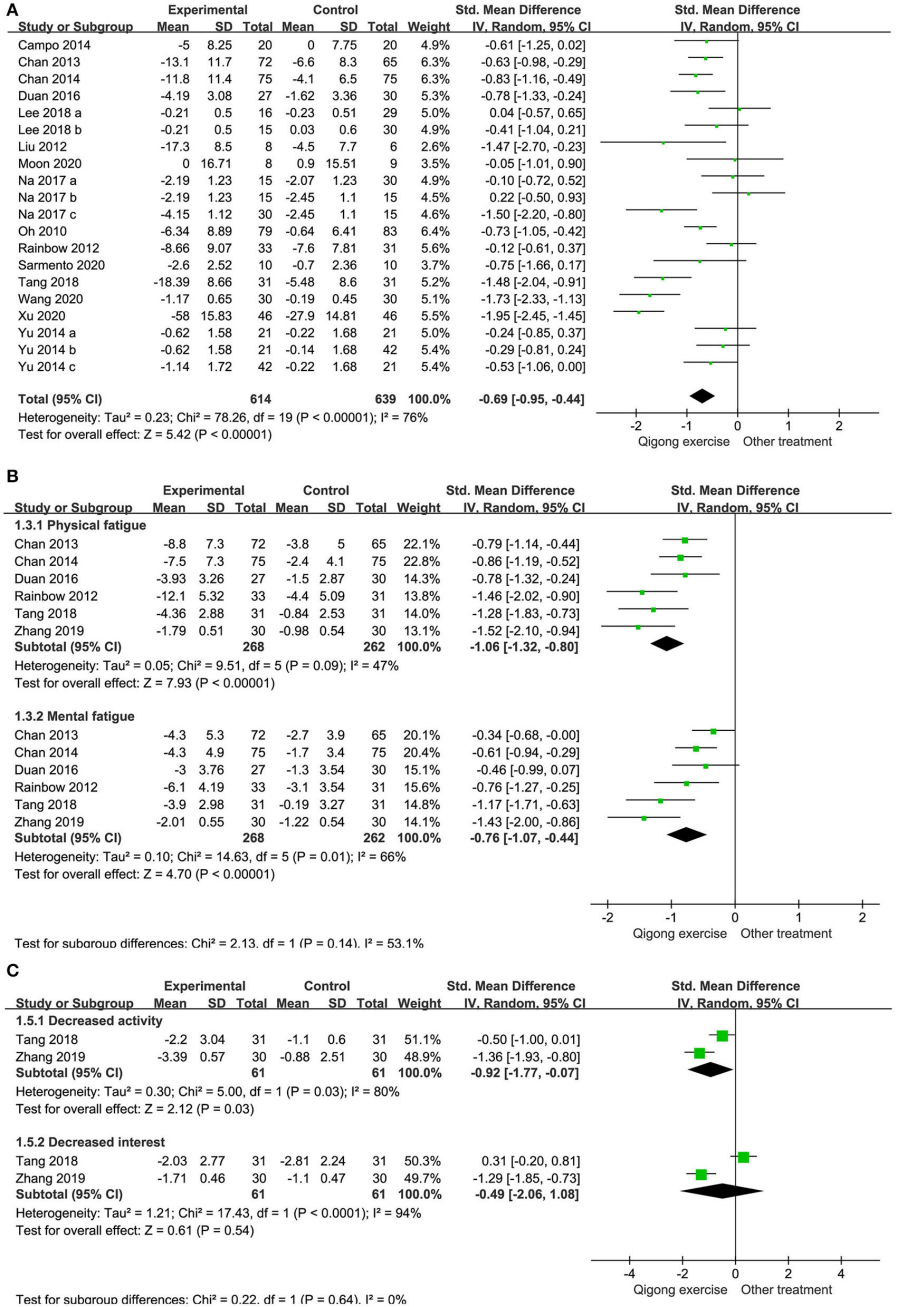
Forest plots illustrating the meta-analysis of outcomes with Qigong exercise (QE) vs. other treatments for fatigue symptoms. The outcomes analyzed were **(A)** total fatigue intensity, **(B)** physical and mental fatigue intensity, and **(C)** decreased activity and interest scale.

Fifteen RCTs involving 1,253 participants reported changes in fatigue intensity. In this cohort, 614 patients performed QE; and 639 patients received other treatments in the control groups. Heterogeneity was evident (I^2^ = 76%, *p* < 0.00001). Analysis of data in the random effect method showed that QE could improve the symptoms of fatigue (SMD −0.69, 95% CI −0.95 to −0.44, *p* < 0.00001; Figure 3A).

##### Physical and Mental Fatigue Intensity

Six studies involving 530 patients were included in our meta-analysis on the efficacy of QE in lowering physical and mental fatigue intensity. The intensity in all the studies was significantly lower with an overall SMD of −1.06 for physical fatigue intensity (95% CI −1.32 to −0.80) and −0.76 for mental fatigue intensity (95% CI −1.07 to −0.44; Figure 3B).

##### Decreased Activity and Interest Scales

Two studies involving 122 patients reported the change in activity and interest levels between baseline and endpoint. In these studies, 61 patients performed QE and 61 served as controls. The results showed that QE could improve the decreased activity levels but not the decreased interest levels. The SMDs were −0.92 and −0.49 for the decreased activity levels (95% CI −1.77 to −0.07, *p* = 0.03) and decreased interest levels (95% CI −2.06 to 1.08, *p* = 0.54; Figure 3C), respectively.

#### Secondary outcomes

##### QOL

**Figure 4:**
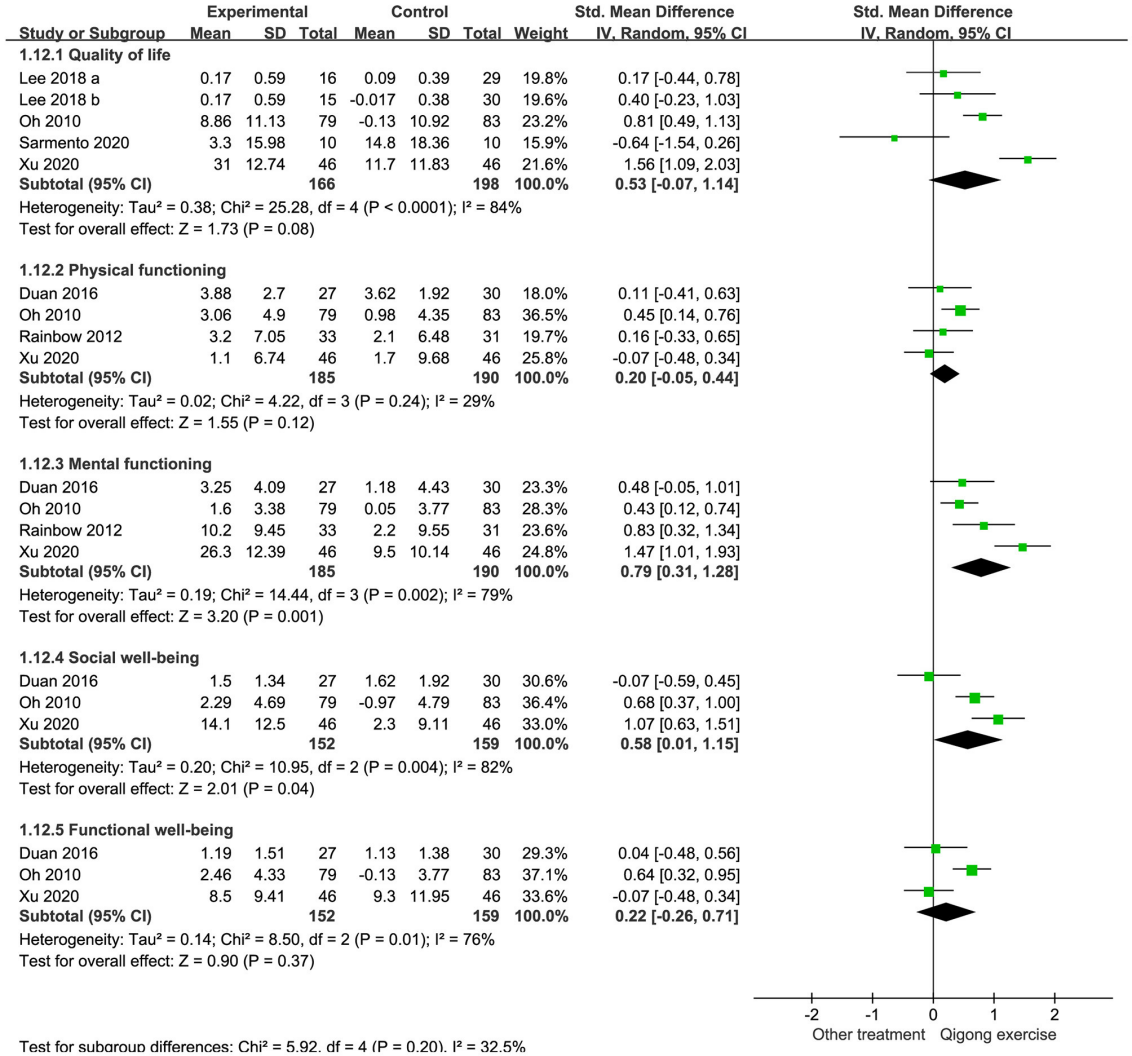
Forest plots illustrating the meta-analysis of outcomes with Qigong exercise (QE) vs. other treatments for quality of life (QOL). The outcomes analyzed were **(A)** QOL scales, **(B)** physical and mental functioning scales, and **(C)** social and functional well-being scales.

Four RCTs reported comparisons of QOL scale scores between patients who performed QE and those who received other treatments. Significant heterogeneity was noted in the enrolled 364 patients (166 in the QE groups and 198 in the control groups) (I^2^ = 84%, *p* < 0.0001). QE could not improve the QOL scale scores (SMD 0.53, 95% CI −0.07 to 1.14; Figure 4A).

Four RCTs reported data on physical and mental functioning scale scores. Heterogeneity was not significant in studies comparing the physical functioning scale scores (I^2^ = 29%, *p* = 0.24) but was significant in those assessing mental functioning (I^2^ = 79%, *p* < 0.001). Data analyses showed that QE could not improve the physical functioning scale scores (SMD 0.20, 95% CI −0.05 to 0.44, *p* = 0.12) but improved the mental functioning scale scores (SMD 0.79, 95% CI 0.31 to 1.28, *p* = 0.001; Figures 4B,C).

Three RCTs compared the social and functional well-being scale scores between the QE and control groups. A combined analysis of 311 patients revealed that QE could improve social well-being (SMD 0.58, 95% CI 0.01 to 1.15, *p* = 0.04) but not functional well-being (SMD 0.22, 95% CI −0.26 to 0.71, *p* = 0.37). Heterogeneities were significant in these two comparisons (I^2^ = 82%, *p* = 0.04 and I^2^ = 76%, *p* = 0.01, respectively; Figures 4D,E).

##### Meta-Regression

Factors examined individually in the meta-regression model of total fatigue intensity included primary diseases (cancer-related fatigue, chronic fatigue syndrome, or other diseases related fatigue), QE type (Baduanjin, other traditional Chinese QE, Wu Xing Ping Heng Gong, “Six healing sound” Qigong, or other self-improving QE), sample size, year of publication, intervention length, or study quality (high or low quality). No significant associations were found between fatigue intensity and the factors (Table 2).

**Table 2:** Meta-regression.

**Variables**	**Coefficient**	**Standard error**	**p**	**95% CI**
Primary diseases	−0.166	0.203	0.413	−0.564 to 0.232
QE type	0.149	0.087	0.088	−0.022 to 0.320
Sample size	−0.003	0.003	0.403	−0.009 to 0.004
Year of publication	−0.053	0.047	0.267	−0.145 to 0.040
Intervention length	−0.025	0.025	0.315	−0.075 to 0.024
Study quality	0.103	0.288	0.722	−0.463 to 0.668

##### Subgroup Analysis

Although meta-regression did not detect significant factors responsible for heterogeneity, we selected different primary disease, QE type, and study quality for subgroup analysis based on the clinical characteristics of the included studies. The results suggested that the QE group with other primary disease-related fatigue showed superior total fatigue intensity improvement compared with that seen in patients with chronic fatigue syndrome and cancer-related fatigue. The Baduanjin QE group presented a trend toward better score improvement when compared with the various types of QE groups. We also found that high-quality studies were better than low-quality studies in demonstrating the benefits of QE for fatigue improvement (Table 3, Supplementary Appendix 3).

**Table 3:** Subgroup analysis.

**Variables**	**Eligible Studies N**	**Eligiblearms N**	**Frequency**		**SMD (95% CI)**	***P***	**Heterogeneity Test**		**Effect Model**
			**QE group N**	**Control group N**			**p**	**I^**2**^, %**	
Different primary diseases									Random
Cancer related fatigue	4	5	176	208	−0.75 (−1.37 to −0.13)	0.02	<0.00001	86%	
Chronic fatigue syndrome	6	10	351	345	−0.50 (−0.75 to −0.24)	0.0001	0.009	59%	
Other diseases related fatigue	5	5	87	86	−1.15 (−1.73 to −0.57)	<0.0001	0.03	62%	
Different types of QE									Random
Baduanjin	4	4	182	182	−1.47 (−2.04 to −0.90)	<0.00001	0.0009	82%	
Other traditional Chinese QE	2	6	144	144	−0.40 (−0.82 to 0.01)	0.06	0.02	64%	
Wu Xing Ping Heng Gong	2	2	105	96	−0.41 (−0.90 to 0.09)	0.10	0.10	64%	
“Six healing sound” Qigong	3	3	26	25	−0.68 (−1.43 to 0.07)	0.07	0.20	38%	
Other self-improving QE	4	5	157	192	−0.56 (−0.83 to −0.28)	<0.0001	0.22	30%	
Different quality of studies									Random
High quality	8	8	350	349	−0.77 (−1.12 to −0.41)	<0.0001	<0.0001	77%	
Low quality	7	12	264	290	−0.65 (−1.02 to −0.27)	<0.01	<0.01	76%	

##### Sensitivity Analysis

Sensitivity analysis was performed in each domain of the primary outcomes to assess the stability of the meta-analysis. When any single study was deleted, the corresponding pooled SMD changed only slightly. The statistical results before and after the deletion were similar, indicating that the stability of the performed meta-analysis was good (Supplementary Appendix 4).

##### Publication Bias

**Figure 5:**
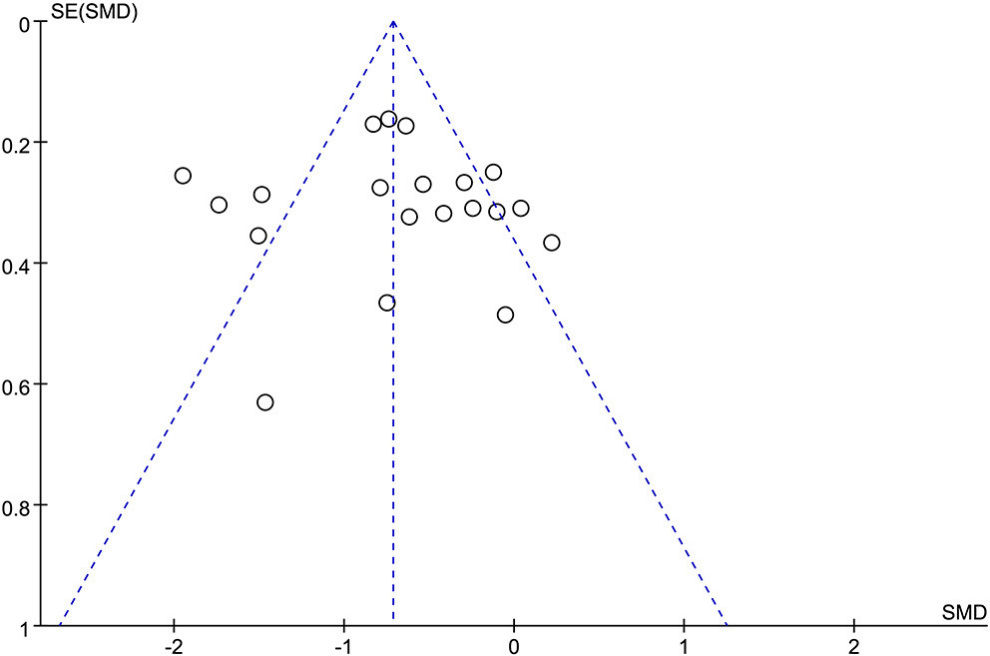
Funnel plot on total fatigue intensity to evaluate the publication bias of the literature. SMD, standardized mean difference.

Publication bias of the literature was tested using the Egger test. A potential publication bias in the meta-analysis on QE intervention was not detected using the funnel plot on total fatigue intensity. The graphical funnel plots of the 15 studies seemed to be symmetrical (Figure 5). We found no evidence of publication bias in the Egger regression test (*t* = 0.27, 95% CI −2.71 to 3.50, *p* = 0.79; Supplementary Appendix 5).

## Discussion

Whether QE is effective in alleviate fatigue is currently unknown. This meta-analysis demonstrated that QE could result in total fatigue intensity improvement in patients with symptoms of fatigue due to various diseases. Moreover, similar results were shown in both physical and mental fatigue intensity and decreased activity levels; however, the change in decreased interest levels was not significant. Moreover, the mental functioning and social well-being scale scores and the pooled data of QOL scale, physical functioning scale, and functional well-being scale scores suggest that QE was not effective.

The marked heterogeneity is a constraint of the analysis of the RCTs included in this study. An important possible reason for the observed heterogeneity is that fatigue symptoms caused by various primary diseases cannot be standardized in the baseline analysis, and differences exist in the individualized assessment scales. The effect of QE on the total fatigue intensity did not vary with the subgroup analysis of the primary diseases. Different types of QE interventions included in this study may also have led to heterogeneity. However, we found that distinguishing between different QE types had little effect on the results of fatigue improvement. In this study, the possible influence of research quality on heterogeneity was also considered, but the results of the subgroup analysis remained unchanged considering the QE effects.

We found that QE did not lead to an improved QOL in patients with fatigue. We hypothesize that this may be due to the overly complex factors that could affect the changes in indicators of QOL. Notably, QE has been effectively used in the rehabilitation of a wide range of diseases (47); it was found to improve the risk factors associated with metabolic syndrome, enhance the QOL of community-dwelling older adults with chronic disease (48), and maintain the health of patients with stable chronic obstructive lung disease (49). The mechanisms by which QE improves fatigue may include positive modulation of immune function (50), balancing the frequency of α1 and α2 waves in different brain regions (51), and increasing serum nitric oxide levels and superoxide dismutase activity to reduce the harmful effects of free radicals in patients (52). However, the mechanism underlying the therapeutic effects requires further studies. The current study revealed certain new negative findings on QE.

Reports on the effects of low-intensity exercise are conflicting. Gendron et al. considered active mind-body movement therapies as an adjunct to, or in comparison with, pulmonary rehabilitation for people with chronic obstructive pulmonary disease; however, the results remain inconclusive (53). Kelley et al. also expressed that a lack of certainty exists with regard to the benefits of exercise on chronic fatigue syndrome in adults (54). The reasons for this contrast could be attributed to the flawed trial protocol design and the poor quality of evidence, which limits their confidence in the observed effects.

We believe that the fatigue in patients may be caused by an intertwining of various diseases and varies in degree depending on age, sex, marital status, occupation, and education, which complicate the analysis of the fatigue symptoms (12). Many hypotheses regarding the pathogenesis of fatigue have been developed; however, no substantial understanding has been garnered so far, which in turn further complicates the treatment (55, 56).

Aerobic exercise, including but not limited to QE, tai chi, walking, and yoga, has been applied as rehabilitation modalities for people with fatigue in primary care, but the abovementioned recent studies did not support these treatments. However, the results of our meta-analysis were mainly positive and the quality of clinical studies in China is also improving. Previous studies often unconsciously ignored the Chinese literature, which was included in our study, since aerobic exercise itself is very popular in China. When we performed data synthesis, we did not eliminate studies based on primary disease, as a result of which we were able to assess a larger number of studies than that done by previous studies. As we sought for an intervention program with a broad clinical effect, our results differ from studies focusing on aerobic exercise.

This study had several limitations. First, the inclusion of multiple different diseases in our study, despite the statistically significant results, is likely to have produced biological bias and is one of the main reasons for the large heterogeneity. Although we performed a meta-regression analysis, it was not successful in identifying the source of heterogeneity in the correlates. The fact that fatigue, as a common symptom in primary care, occurs in different diseases is the reason for the inevitable heterogeneity of this study. We therefore performed subgroup analysis according to different primary diseases, which still showed positive results. Second, the determination of fatigue is very subjective and this has led to differences in the evaluation criteria across the included literatures. Finally, the lack of uniform QE types and intervention length may have contributed to the bias in the overall results of this study. Moreover, if individual patient data are accurate and detailed, subgroup analyses can be adjusted for age, race, and geographic location. Therefore, the statistical power of the relevant sections was influenced to a certain extent. These limitations can be overcome by conducting larger and higher quality clinical trials so that we can standardize the details of the specific diseases and QE intervention conditions.

## Conclusion

Our meta-analysis demonstrated that QE was able to improve fatigue symptoms, including total fatigue intensity and physical and mental fatigue intensity. QE for fatigue in patients or the general population has not been well investigated, with only a few publications regarding this common but under-diagnosed clinical problem. Future well-designed, high-quality studies with larger sample sizes are warranted.

## Data Availability Statement

The original contributions presented in the study are included in the article/Supplementary Material, further inquiries can be directed to the corresponding author.

## Author Contributions

DS: conceptualization, supervision, writing — review, and editing. RW: data curation, funding acquisition, validation, and writing — original draft. RW and XH: methodology. XH and YW: software. All authors contributed to the article and approved the submitted version.

## Conflict of Interest

The authors declare that the research was conducted in the absence of any commercial or financial relationships that could be construed as a potential conflict of interest.
