# Supplementary figures and images for: Efficacy of Qigong Exercise for Treatment of Fatigue: A Systematic Review and Meta-Analysis
Source: Front Med (Lausanne). 2021 Jun 22;8:684058. doi: 10.3389/fmed.2021.684058 (PMC8257957; doi:10.3389/fmed.2021.684058)

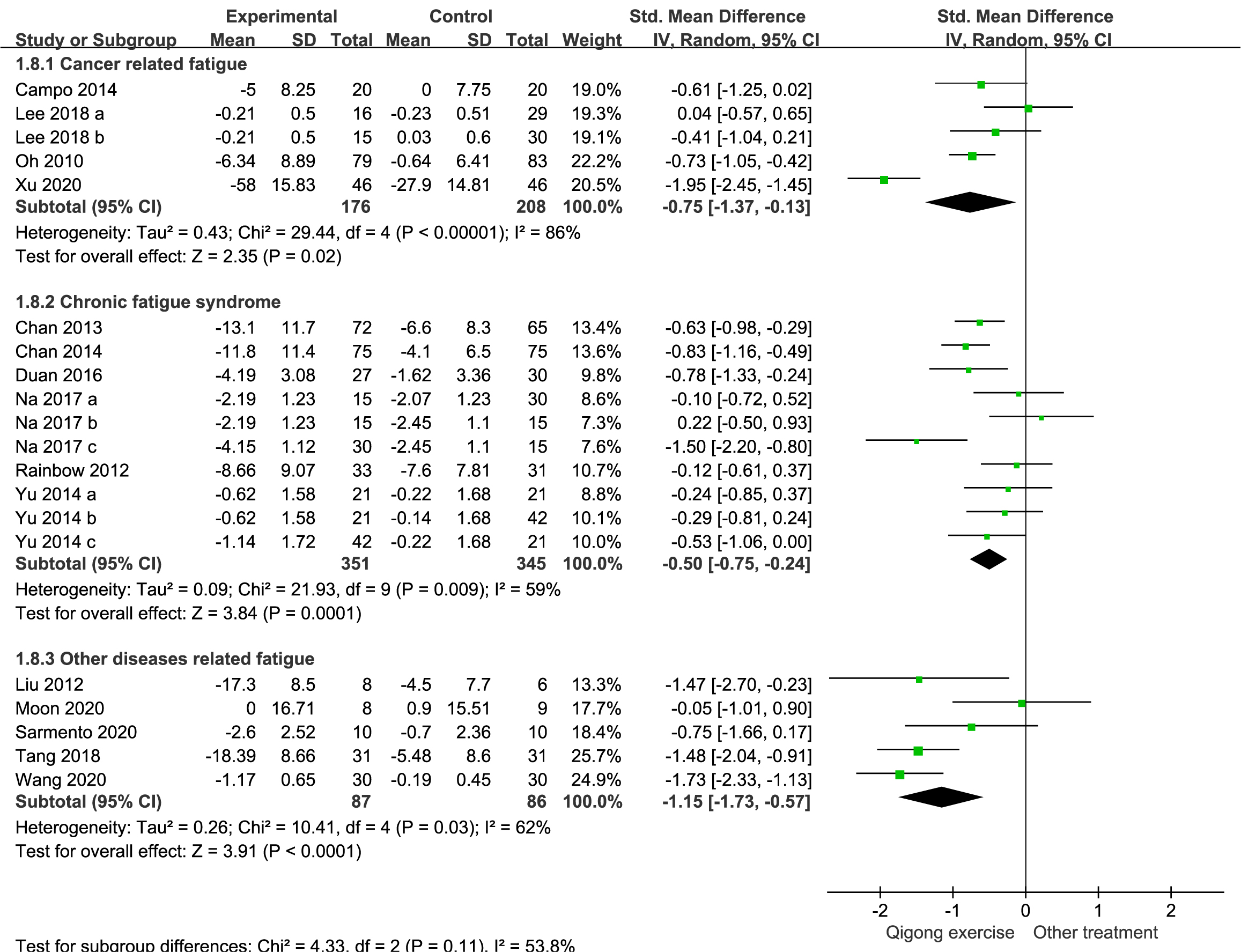

Supplement: Supplementary file 1 [file Image_1.JPEG]

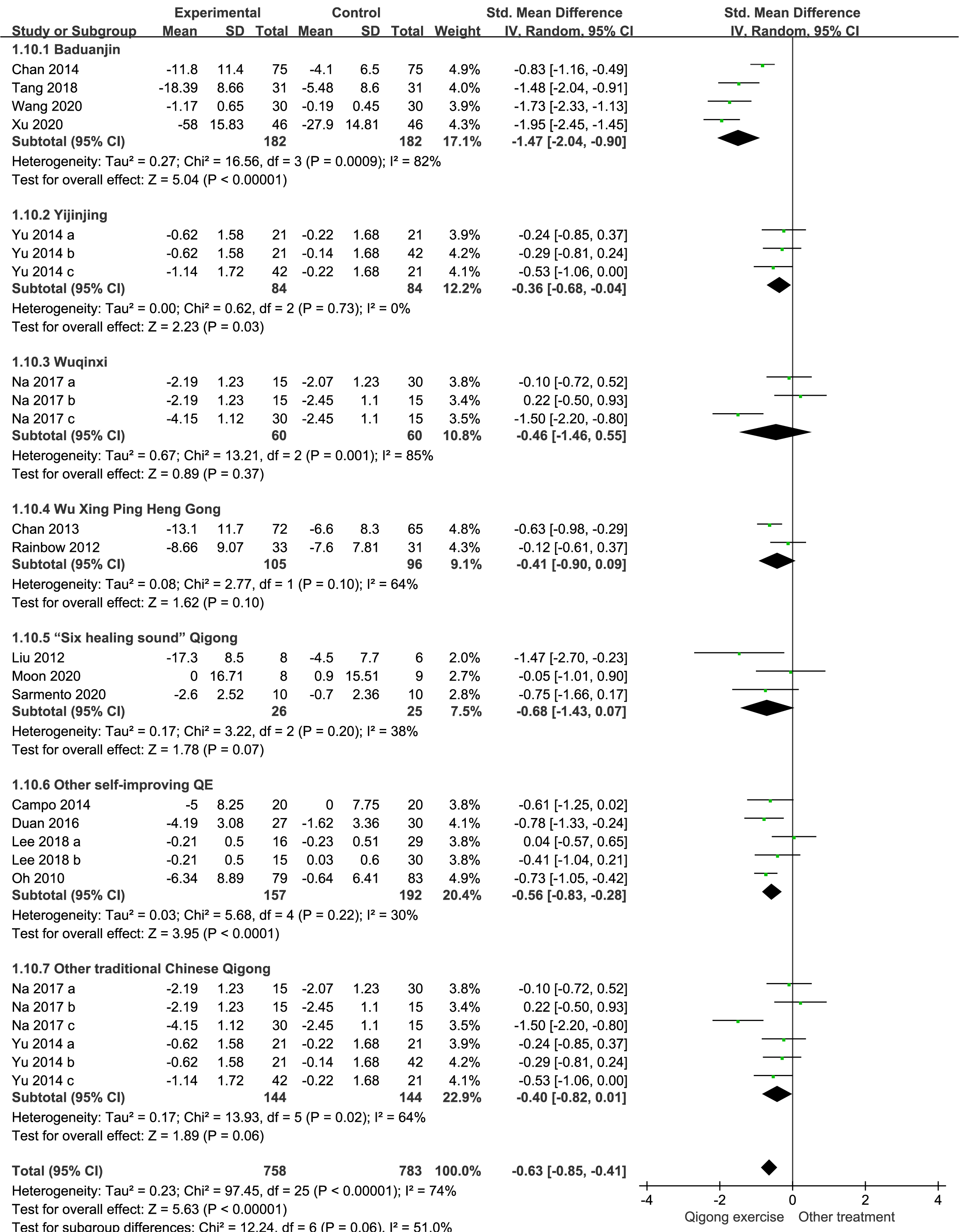

Supplement: Supplementary file 2 [file Image_2.JPEG]

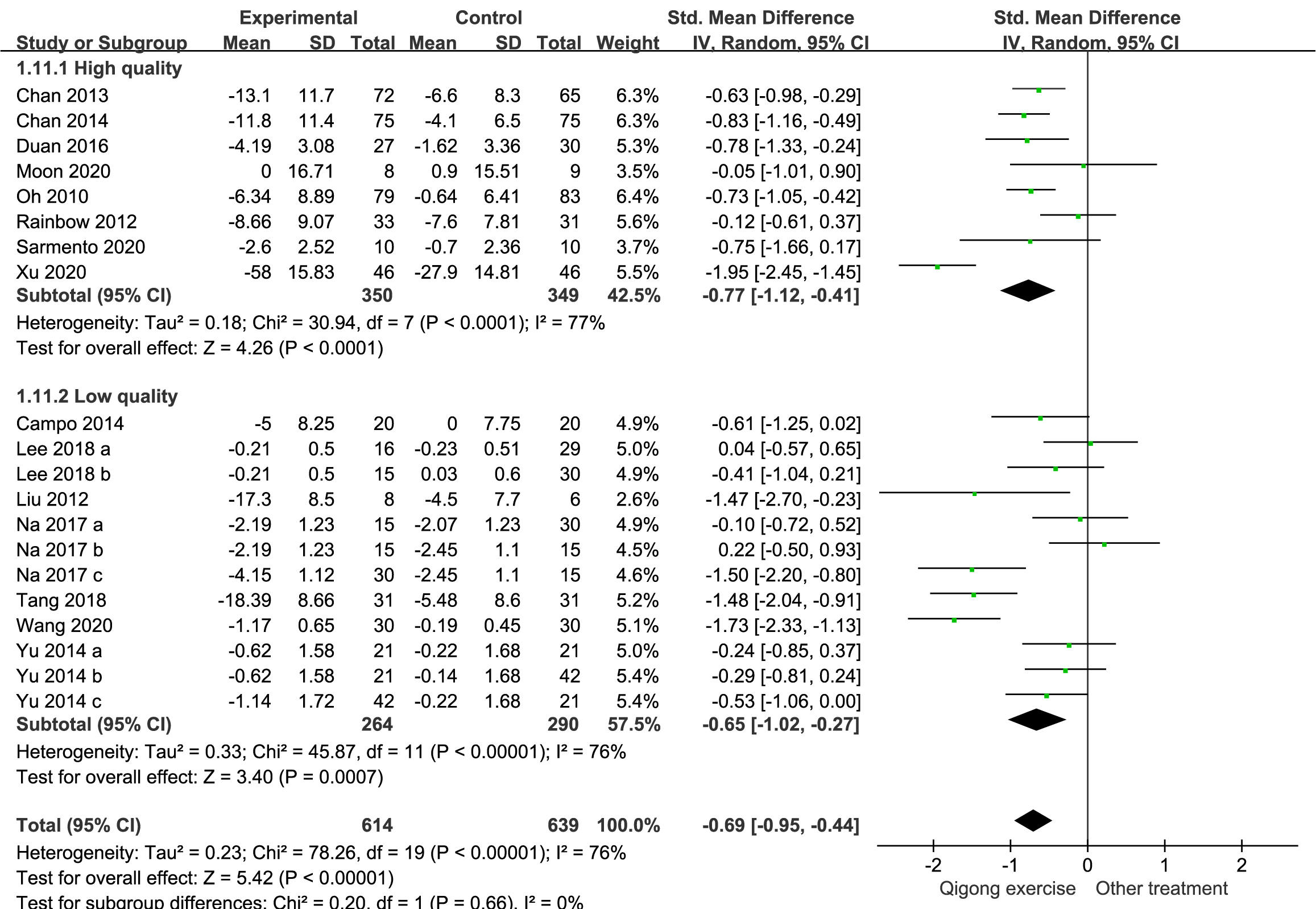

Supplement: Supplementary file 3 [file Image_3.JPEG]

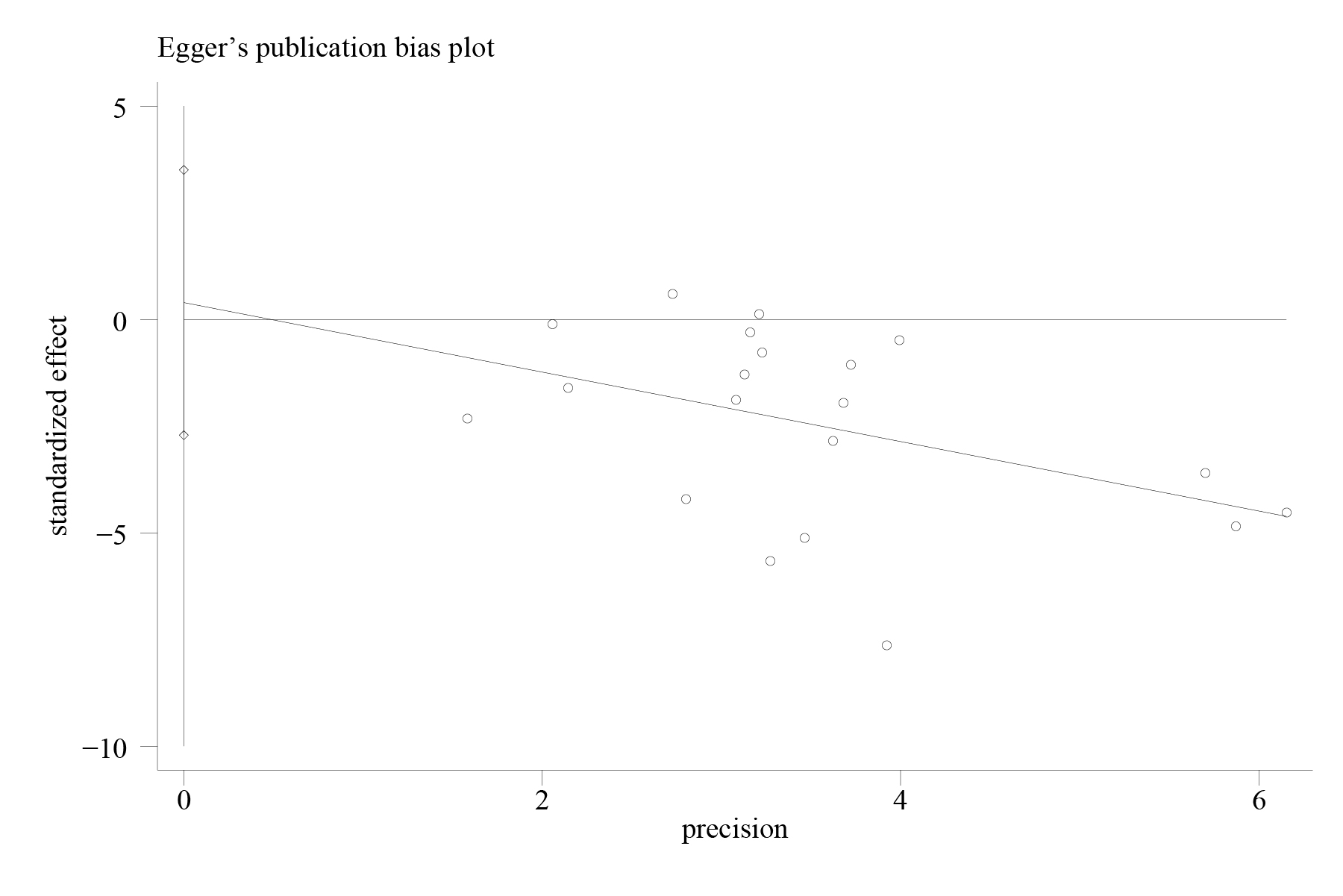

Supplement: Supplementary file 4 [file Image_4.JPEG]
