# Supplementary material for: Efficacy of Qigong Exercise for Treatment of Fatigue: A Systematic Review and Meta-Analysis
Source: Front Med (Lausanne). 2021 Jun 22;8:684058. doi: 10.3389/fmed.2021.684058 (PMC8257957; doi:10.3389/fmed.2021.684058)
Supplement: Supplementary file 5 [file Table_1.DOCX]

**Supplementary appendix 4.** Sensitivity analysis of total fatigue intensity (Omitting a Single RCT).

|  | SMD (95% CI) | p | Heterogeneity Test | |
| --- | --- | --- | --- | --- |
|  |  |  | p | I^2^, % |
| All studies | -0.69 (-0.95 to -0.44) | < 0.00001 | < 0.00001 | 76% |
| Selected study omitted | | | | |
| Campo, 2014 | -0.70 (-0.96 to -0.44) | < 0.00001 | < 0.00001 | 77% |
| Chan, 2013 | -0.72 (-0.85 to -0.60) | < 0.00001 | < 0.00001 | 77% |
| Chan, 2014 | -0.70 (-0.82 to -0.57) | < 0.00001 | < 0.00001 | 77% |
| Duan, 2016 | -0.71 (-0.83 to -0.59) | < 0.00001 | < 0.00001 | 77% |
| Lee, 2018 a | -0.74 (-0.86 to -0.62) | < 0.00001 | < 0.00001 | 75% |
| Lee, 2018 b | -0.72 (-0.84 to -0.60) | < 0.00001 | < 0.00001 | 77% |
| Liu, 2012 | -0.71 (-0.82 to -0.59) | < 0.00001 | < 0.00001 | 77% |
| Moon, 2020 | -0.72 (-0.84 to -0.60) | < 0.00001 | < 0.00001 | 76% |
| Na, 2017 a | -0.74 (-0.86 to -0.62) | < 0.00001 | < 0.00001 | 76% |
| Na, 2017 b | -0.74 (-0.86 to -0.62) | < 0.00001 | < 0.00001 | 75% |
| Na, 2017 c | -0.69 (-0.81 to -0.57) | < 0.00001 | < 0.00001 | 75% |
| Oh, 2010 | -0.71 (-0.84 to -0.58) | < 0.00001 | < 0.00001 | 77% |
| Rainbow, 2012 | -0.75 (-0.87 to -0.63) | < 0.00001 | < 0.00001 | 75% |
| Sarmento, 2020 | -0.71 (-0.83 to -0.59) | < 0.00001 | < 0.00001 | 77% |
| Tang, 2018 | -0.68 (-0.80 to -0.56) | < 0.00001 | < 0.00001 | 75% |
| Wang, 2020 | -0.67 (-0.79 to -0.55) | < 0.00001 | < 0.00001 | 73% |
| Xu, 2020 | -0.64 (-0.76 to -0.52) | < 0.00001 | < 0.00001 | 66% |
| Yu, 2014 a | -0.73 (-0.85 to -0.61) | < 0.00001 | < 0.00001 | 76% |
| Yu, 2014 b | -0.74 (-0.86 to -0.61) | < 0.00001 | < 0.00001 | 76% |
| Yu, 2014 c | -0.72 (-0.84 to -0.60) | < 0.00001 | < 0.00001 | 77% |
